# Determining factors affecting the acceptability of spirometry: A survey study in a tertiary chest diseases center

**DOI:** 10.5578/tt.20239708

**Published:** 2023-09-22

**Authors:** M.O. Güçsav, G. POLAT, D. Serçe Unat, E. Bayramiç, E.S. Dikmentepe Yılmaz

**Affiliations:** 1 Department of Chest Diseases, Bakırçay University Faculty of Medicine, İzmir, Türkiye; 2 Clinic of Chest Diseases, Health Sciences University Dr. Suat Seren Chest Diseases and Chest Surgery Training and Research Hospital, İzmir, Türkiye; 3 Clinic of Chest Diseases, Kemalpaşa Hospital, İzmir, Türkiye; 4 Clinic of Chest Diseases, Çorum Chest Diseases Hospital, Çorum, Türkiye

**Keywords:** spirometry, acceptability, anxiety, experience, asthma

## Abstract

**ABSTRACT:**

Determining factors affecting the acceptability of spirometry: A survey study in a tertiary chest diseases center

**Introduction:**

Unlike other laboratory tests, spirometry requires the participant’s full compliance with the maneuvers in the test for an acceptable test result. In this study, we aimed to determine the suitability of spirometric tests regarding acceptability and the factors associated with acceptability.

**Materials and Methods:**

Before the test, our 15-scale questionnaire, prepared by us in the respiratory function laboratory, was applied to the participants who requested spirometric examination in our hospital. Afterwards, patients were subjected to spirometric analysis. Spirogram results of the participants were evaluated by four clinicians who were experts in the field based on the acceptability criteria in the American Thoracic Society and European Respiratory Society Spirometry Standardization Guidelines. Participants were divided into two groups as those who met the acceptability criteria and those who did not. Both groups were compared regarding demographic data, comorbidities, education levels, and questions in the questionnaire.

**Results:**

The acceptability spirometry rate was 71.2%. The most common error among those who could not perform an acceptable test was the inability to complete the expiratory time to the time that would create a plateau, with 37.3%. Education level and acceptability of spirometry were not related (p= 0.228). Asthma was statistically significantly higher in the group that performed acceptable spirometry (p= 0.049). Acceptable spirometry rate was statistically significantly higher in the participants who had previously performed spirometric tests compared to the other group (p< 0.001). The test success of the participants who did not have success anxiety about the test was significantly higher than the other group (p= 0.033).

**Conclusion:**

Reduction of participants’ anxiety and repetitive testing increases test acceptability. For this reason, in our clinical practice, we recommend that people who want a spirometry test relieve their anxiety about the test and repeat the test in unacceptable tests.

## Abstract (tr)

**ÖZ:**

Spirometrinin kabul edilebilirliğini etkileyen faktörlerin belirlenmesi: Üçüncü basamak bir göğüs hastalıkları merkezinde anket çalışması

**Giriş:**

Spirometri diğer laboratuvar testlerinden farklı olarak kabul edilebilir bir test sonucu için katılımcının testteki manevralara tam uyumunu gerektirir. Çalışmamızda spirometrik testlerin kabul edilebilirlik açısından uygunluğu ve kabul edilebilirlik ile ilişkili olan faktörlerin belirlenmesi amaçlanmıştır.

**Materyal ve Metod:**

Hastanemizde spirometrik tetkik istenen katılımcılara test öncesinde solunum fonksiyon laboratuvarında tarafı- mızca hazırlanan 15 ölçeklik anketimiz uygulandı. Sonrasında hastalar spirometrik analize tabi tutuldu. Katılımcıların spirogram sonuçları alanında uzman dört klinisyen tarafından ATS/ERS Spirometri Standardizasyon Rehberi’nde yer alan kabul edilebilirlik kriterleri baz alınarak değerlendirildi. Katılımcılar kabul edilebilirlik kriterlerini karşılayanlar ve karşılayamayanlar olarak iki gruba ayrıldı. Her iki grup demografik veriler, ek hastalıklar, eğitim düzeyleri ve ankette yer alan sorular açısından karşılaştırıldı.

**Bulgular:**

Kabul edilebilirlik spirometri oranı %71,2 idi. Kabul edilebilir test yapamayanlarda en sık hata %37,3 ile ekspiryum süresinin plato oluşturacak süreye tamamlanamamasıydı. Eğitim düzeyi ile spirometri kabul edilebilirliği ilişkili değildi (p= 0,228). Kabul edilebilir spirometri yapan grupta astım istatistiksel olarak anlamlı düzeyde fazlaydı (p= 0,049). Daha önce spirometrik test yapan katılımcılarda kabul edilebilir spirometri oranı diğer gruba göre istatistiksel olarak anlamlı düzeyde yüksekti (p< 0,001). Test hakkında başarı anksiyetesi olmayan katılımcılarda test başarısı diğer gruba göre anlamlı düzeyde yüksekti (p= 0,033).

**Sonuç:**

Katılımcının anksiyetesinin azalması ve tekrarlayan test uygulaması test kabul edilebilirliğini arttırmaktadır. Bu nedenle klinik pratiğimizde spirometri tetkiki istediğimiz kişilerin test hakkındaki anksiyetesini gidermeyi ve kabul edilebilir olmayan testlerde testin tekrarlanmasını önermekteyiz.

## INTRODUCTION

Spirometry is a physiological test widely used to evaluate respiratory functions today, based on measuring air volume and ventilatory flow created by the person in inspiration and expiration ( 1 , 2 ). This test is essential in diagnosing many lung diseases, especially chronic obstructive pulmonary disease (COPD) and asthma ( 2 ). Unlike other laboratory tests, the participant’s full compliance with the maneuvers in the test is required ( 3 ). The fact that the demographic characteristics of the participants, their education levels, or the level of perception of the test are different, and the variability of their compliance with the health worker who administers the test causes the test results to be variable.

Studies show that the range of expected values for populations can be narrowed, and abnormal test results can be detected more accurately by standardizing measurement values ( 11 ). For this reason, a statement was first published by the American Thoracic Society (ATS) in 1979 to provide standardization in evaluating spirometric examinations ( 4 ). Over the years, these criteria have been updated many times ( 1 , 2 ).

Some studies examine the factors affecting the acceptability of spirometric measurement in children, young and elderly populations, and similar factors ( 5 , 6 , 7 ). However, there are not enough studies examining the effects of parameters such as compliance with the rules that participants must comply with before the test. In our study, with the help of a questionnaire, many parameters such as the level of knowledge of the participants about the procedure, their anxiety status, and how much they complied with the rules to be followed before the spirometric measurement were evaluated, and it was aimed to investigate the relationship between these parameters and test acceptability.

## MATERIALS and METHODS

This study was a questionnaire-mediated crosssectional study in a tertiary chest diseases center. For this purpose, patients admitted for spirometry examination due to diagnosis, follow-up, disability, and preoperative evaluation between 01.09.2021 and 31.09.2022 were included. The research was carried out in accordance with the 1964 Declaration of Helsinki and its subsequent amendments after it was approved by the ethics committee of our institution (Approval no: 2021/32-39 and date: 02.07.2021).

Inclusion criteria for the study were determined as patients between 17-80 years of age who gave informed consent to the study. Among these people those who were pregnant, those with a history of COVID-19 in the last one month, those with thorax/ extremity deformities that would limit spirometry maneuvers, those who were asked to be tested despite having any of the relative contraindications, and those who did not answer more than one of the questions in the questionnaire were excluded from the study.

**Figure 1:**
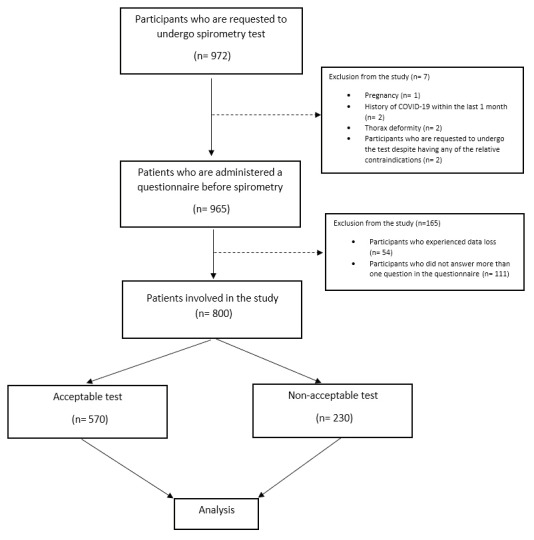
Flow chart.

After the questionnaire, the participants were taken to the laboratory for spirometry examination. Patients were informed verbally before the test as in our daily practice. No additional visual or written material was used to inform any patient. Firstly, the height and weight of the participants were measured. Then, the participants were subjected to spirometric analysis with a Zan (Germany) brand spirometry device. All measurements were conducted with the same device. Test manoeuvres were performed under the guidance of technicians. For the study, two technicians who have been working in the PFT laboratory for more than one year were assigned. These technicians completed standardised courses before working in our laboratory. Before starting the study, the technicians’ approach before and during the test was evaluated in 10 patients not included in the study. The evaluation was based on the criteria in the “Turkish Thoracic Society Consensus Report: Interpretation of Spirometry” ( 8 ). Before initiating the maneuvers, attention was paid to ensure that the participants were sitting, their noses were closed with a nose clip, and the mouthpiece was placed in the mouth in a way that would not leak. Each participant was asked to perform at least three maneuvers. The number of maneuvers of the participants who could not perform acceptable spirometry in all three maneuvers was completed to a maximum of five. The spirogram results of the participants were evaluated by four clinicians who are experts in the field based on the acceptability criteria in the ATS/ERS Spirometry Standardization Guide 2019 ( 2 ). Accordingly, the participants were divided into two groups: those who met the acceptability criteria and those who did not. Participants in both groups were compared regarding demographic data, comorbidities, education levels, and questions in the questionnaire. Obtained data were analyzed statistically ( Figure 1 ).

Data in the study were analyzed via IBM SPSS version 26.0 package program (IBM Corp., Armonk, NY). The Kolmogorov-Smirnov test was used to evaluate whether all variables fit the normal distribution. Descriptive data were presented as mean ± standard deviation (SD) or median (minimum– maximum). Categorical variables were evaluated with the Chi-square test, and continuous variables were evaluated with the Student’s t-test. Values below p< 0.05 were considered statistically significant in the analyses.

## RESULTS

A total of 972 participants who met the inclusion criteria were evaluated within the scope of this research. After eliminating 172 individuals who did not meet the inclusion criteria, the remaining 800 participants were enrolled. Spirometric testing of 230 participants (28.8%) did not meet the acceptability criteria. The most common error among those who could not perform an acceptable test was the inability to complete the expiratory time to the time that would create a plateau (37.3%). This was followed by cough or glottic leak finding in the expiratory ring, with a rate of 23.4%. The extrapolation volume was less than 100 mL in 47 participants (11.9%), and 45 participants (11.4%) did not meet at least one of the forced end-expiratory indicators. Time to peak current was less than 120 ms in 37 participants (9.4%). The number of participants who terminated the test early was 26 (6.6%). There was no obstruction or leak in the spirometer or mouthpiece in any spirograms. No zero flow reference point calibration error was detected in any measurements ( Table 1Table 1 ).

Regarding sex, 53% of the study population were males and 47% were females. Mean age of the participants was 49.9 ± 16.8 years. Only 9.3% of the participants were severely obese (Body mass index ≥35 kg/m2), and 17.3% were obese (body mass index 30-35 kg/m2). Hypertension was the most common comorbid disease (23.8%), followed by asthma and COPD (22.4% vs. 16.4%). Tobacco consumption could be elaborated as 39.1% of the participants were active smokers, 29% were ex-smokers, and 31.9% were non-smokers. When their educational status was examined, it was seen that 39 (4.9%) of the participants were illiterate= 259 (32.5%) were primary school, 120 (15.1%) were secondary school, 213 (26.7%) were high school, and 140 (17.5%) were university graduates, and 26 (3.3%) had a graduate degree.

The distribution of the answers to the survey questions by the entire participant population is shown in Table 2 .

Groups of participants with and without acceptable spirometric measurements were compared regarding demographic data. No correlation was found between age, sex, body mass index, smoking, and acceptability of spirometry (p= 0.515, p= 0.216, p= 0.978, and p= 0.062). While the highest acceptability rate in spirometry was seen in university (78.6%) and graduate students (76.9%), spirometry’s acceptability decreased as the education level decreased. However, this difference was not statistically significant (p= 0.228). When both groups were compared regarding comorbid diseases, asthma disease was statistically significantly higher in the group that performed acceptable spirometry (p= 0.049). There was no statistically significant difference between the groups in other comorbid diseases.

The acceptable spirometry rate was statistically significantly higher in the participants who had previously performed spirometric tests compared to the other group (p< 0.001). However, no correlation was found between the number of tests performed by the participants and test acceptability (p= 0.937). The presence of anxiety was assessed according to the response to the question “Are you afraid you won’t be able to do the test successfully?” in the questionnaire. Accordingly, the test success of the participants who were anxious that they would not be able to perform the test successfully was significantly lower compared to the group who were not anxious about this issue (p= 0.033). Informing the doctor before the test did not affect test acceptability (p= 0.423). Similarly, there was no effect of being informed by non-health personnel on test acceptability (p= 0.457). The comparison of the groups in terms of their responses to other survey parameters is shown in Table 3 .

**Table 1:** Analysis of acceptability criteria

Acceptability Criteria	n (%)
Failure to reach plateau in expiration time	147 (37.3)
Cough/glottic leak	92 (23.4)
Extrapolation volume of FVC≤ 5% or 0.100 L	47 (11.9)
Change in last one second of expiration ≤0.025 L	45 (11.4)
Time to reach peak flow <120 ms	37 (9.4)
Early termination of the test	26 (6.6)
Obstruction or leak in mouthpiece	0 (0.0)
Incorrect zero flow setting	0 (0.0)
Total*	394 (100)

*Multiple reasons were marked in participants whose spirometry was not acceptable.

**Table 2:** Distribution of the responses to survey questions

Variable		n (%)
1. Is this your first time having a pulmonary function test? (n= 800)	No	469 (58.6)
	Yes	331 (41.4)
2. If no, how many times have you had a pulmonary function test before? (n= 469)	1-5	350 (74.7)
	5-10	74 (15.7)
	>10	45 (9.6)
3. How much information do you think your doctor gave you about the test before the pulmonary function test? (n= 798)	None	124 (15.5)
	Insufficient	76 (9.5)
	Sufficient	598 (74.9)
4. Did you receive any information about the test from another patient or someone you know who has already had the test before your pulmonary function test? (n= 798)	No	444 (55.6)
	Yes	354 (44.4)
5. Do you know why your doctor asked you to have this test? (n= 798)	No	89 (11.2)
	Yes	709 (88.8)
6. Do you know the benefits of the test for diagnosis? (n= 798)	No	309 (38.7)
	Yes	489 (61.3)
7. Do you think you will experience pain or discomfort during the test? (n= 798)	No	711 (89.1)
	Yes	87 (10.9)
8. Are you afraid you won’t be able to do the test successfully? (n= 798)	No	676 (84.7)
	Yes	122 (15.3)
9. Would you like to be informed in detail about your test results? (n= 798)	No	205 (25.7)
	Yes	593 (74.3)
10. Have you smoked within the last hour? (n= 797)	No	670 (84.1)
	Yes	127 (15.9)
11. Have you consumed alcohol within the last 8 hours? (n= 798)	No	793 (99.4)
	Yes	5 (0.6)
12. Have you had a heavy/fatty meal or eaten too much within the last 2 hours? (n= 798)	No	754 (94.5)
	Yes	44 (5.5)
13. Have you engaged in heavy exercise or a strenuous activity within the last hour? (n= 797)	No	776 (97.4)
	Yes	21 (2.6)
14. Have you taken any bronchodilator medication within the last 24 hours? (n= 798)	No	653 (81.8)
	Yes	145 (18.2)
15. Are you wear uncomfortable clothing on the test day that was restrictive, such as a corset, tight shirt, or tight pants? (n= 799)	No	734 (91.9)
	Yes	65 (8.1)

**Table 3:** Comorbid conditions accompanying patients

		Acceptability of spirometry		p
		No (n, %)	Yes (n, %)	
1. Is this your first time having a pulmonary function test?		(n= 230)	(n= 570)	<0.001
	No	105 (45.6)	364 (63.8)	
	Yes	125 (54.4)	206 (36.2)	
2. If no, how many times have you had a pulmonary function test before?		(n= 230)	(n= 570)	0.937
	1-5	79 (34.4)	272 (47.7)	
	5-10	16 (6.7)	58 (10.2)	
	>10	10 (4.5)	34 (5.9)	
3. How much information do you think your doctor gave you about the test before the pulmonary function test?		(n= 229)	(n= 569)	0.423
	None	41 (17.9)	83 (66.9)	
	Insufficient	19 (8.3)	57 (75.0)	
	Sufficient	169 (73.8)	429 (71.7)	
4. Did you receive any information about the test from another patient or someone you know who has already had the test before your pulmonary function test?		(n= 229)	(n= 567)	0.457
	No	121 (52.8)	323 (57.0)	
	Yes	108 (47.2)	244 (43.0)	
5. Do you know why your doctor asked you to have this test?		(n= 230)	(n= 568)	0.510
	No	23 (10)	207 (90)	
	Yes	66 (11.6)	502 (88.4)	
6. Do you know the benefits of the test?		(n= 230)	(n= 568)	0.527
	No	93 (40.4)	137 (59.6)	
	Yes	216 (38.0)	352 (62.0)	
7. Do you think you will experience pain or discomfort during the test?		(n= 230)	(n= 568)	0.217
	No	200 (86.9)	30 (13.1)	
	Yes	511 (90.0)	57 (10.0)	
8. Are you afraid you won’t be able to do the test successfully?		(n= 230)	(n= 568)	0.033
	No	185 (80.4)	45 (19.6)	
	Yes	491 (86.4)	77 (13.6)	
9. Would you like to be informed in detail about your test results?		(n= 230)	(n= 568)	0.732
	No	61 (26.5)	169 (73.5)	
	Yes	144 (25.4)	424 (74.6)	
10. Have you smoked within the last hour?		(n= 229)	(n= 568)	0.337
	No	197 (86.0)	32 (14.0)	
	Yes	473 (83.3)	95 (16.7)	
11. Have you consumed alcohol within the last eight hours?*		(n= 229)	(n= 569)	-
	No	229 (100)	0 (0)	
	Yes	564 (99.1)	5 (0.9)	
12. Have you had a heavy/fatty meal or eaten too much within the last two hours?		(n= 229)	(n= 569)	0.054
	No	222 (97.0)	7 (3.0)	
	Yes	532 (93.5)	37 (6.5)	
13. Have you engaged in heavy exercise or a strenuous activity within the last hour?		(n= 229)	(n= 568)	0.138
	No	226 (98.7)	3 (1.3)	
	Yes	550 (96.7)	18 (3.3)	
14. Have you taken any bronchodilator medication within the last 24 hours?		(n= 229)	(n= 569)	0.122
	No	195 (85.2)	34 (14.8)	
	Yes	458 (80.5)	111 (19.5)	
15. Are you wear uncomfortable clothing on the test day that was restrictive, such as a corset, tight shirt, or tight pants?		(n= 230)	(n= 569)	0.939
	No	211 (91.7)	19 (8.3)	
	Yes	523 (91.9)	46 (8.1)	

*There couldn’t be an evaluation due to insufficient number of participants in an eye.

## DISCUSSION

In this study, the acceptable rate of spirometric testing was found to be 71.2% in adults over the age of 18. In the literature, it is seen that the acceptability rates vary according to the society in which the studies are conducted. In a study conducted in our country, the rate of tests meeting the acceptability criteria was 62.4% ( 9 ). In the study by Li et al. in a large population in China, this rate was 98% ( 10 ). In a study conducted in Italy on the population over 65 years of age, it was observed that the acceptability rates were around 80% ( 11 ). When the studies conducted in our country were compared with other studies, it was seen that the acceptability rates of spirometry were behind those of other developed world countries.

Studies show that repetition is one of the essential steps in learning ( 12 ). Spirometric test is an examination that takes time to learn because it contains maneuvers that require compliance with commands. In our study, the acceptability of spirometry was significantly higher in participants who had previously undergone spirometry testing than those who experienced it for the first time. Therefore, as the number of spirometric test applications increases, test acceptability increases.

In our study, test acceptability of the participants with a diagnosis of asthma was significantly higher than the other participants. However, although this was statistically significant, it was not clinically significant. This was because participants diagnosed with asthma had more spirometric tests in the past, had a higher education level, and were younger in age.

Studies show that parameters such as forced vital capacity (FVC), vital capacity (VC), and forced expiratory volume in one second (FEV1) are measured lower in people with anxiety compared to those without anxiety ( 13 ). However, Makonga-Braaksma et al., in their study on the effect of anxiety on test acceptability, have found no correlation between pretest anxiety and acceptability ( 14 ). Contrary to this study, in our study, spirometry acceptability was significantly lower in participants with pretest success anxiety. Anxiety reduces success in many cognitive and physical functions, which is a widely known condition. For this reason, a spirometry examination, which requires full compliance with the commands and a challenging effort, is considered an expected situation for people under anxiety to have low acceptable spirometry rates. We think that there are studies in the literature that present data contrary to our study related to the fact that the anxiety levels of the participants included in the study cannot be classified.

In our study, no relation was found between the level of information and test acceptability. However, we could not detect this relation because of the technician’s support during the test. In support of this, studies in the literature show the effect of coaching in tests requiring effort on test success ( 15 , 16 ).

In our study, although acceptable spirometry rates increased as education level increased, no statistical relationship was found between education level and spirometry acceptability. Although there is no precise data in the literature on this subject, studies show that the duration of education is related to the acceptability of spirometry ( 11 ). According to OECD data, 39% of the people in Türkiye need help understanding what they read regardless of education level. In addition to this, the short examination time per patient disrupts the relation between education level and test acceptability ( 17 ).

In the spirometry standardization guide prepared by ATS/ERS, recommendations to be followed before testing are stated ( 2 ). Among these recommendations are studies investigating the effects of smoking, heavy and fatty diet, alcohol use, and exercise on spirometric parameters ( 18 , 19 ). Our study investigated the effect of compliance with these recommendations on test acceptability. However, no statistically significant effect was found. Our work in this area contributes to the literature.

As a result, decreasing the participant’s anxiety and repetitive testing increase test acceptability. For this reason, in our clinical practice, we recommend that people who want a spirometry test relieve their anxiety about it and repeat it in unacceptable tests.

## Ethical Committee Approval

This study was approved by the Health Sciences University İzmir Dr. Suat Eren Chest Diseases and Surgery Training and Research Clinical Research Ethics Committee (Decision no: 2021/32-39, Date: 02.07.2021).

## CONFLICT of INTEREST

The authors declare that they have no conflict of interest.

## AUTHORSHIP CONTRIBUTIONS

Concept/Design: MOG, GP

Analysis/Interpretation: All of authors

Data acqusition: All of authors

Writing: MOG, DSU

Clinical Revision: All of authors

Final Approval: All of authors
